# Selective Inhibition of Retinal Angiogenesis by Targeting PI3 Kinase

**DOI:** 10.1371/journal.pone.0007867

**Published:** 2009-11-17

**Authors:** Yolanda Alvarez, Olaya Astudillo, Lasse Jensen, Alison L. Reynolds, Nora Waghorne, Derek P. Brazil, Yihai Cao, John J. O'Connor, Breandán N. Kennedy

**Affiliations:** 1 UCD Schools of Biomolecular and Biomedical Sciences, UCD Conway Institute, University College Dublin, Dublin, Ireland; 2 Institute for Microbiology Tumor and Cell Biology, Karolinska Institute, Stockholm, Sweden; 3 Centre for Vision and Vascular Science, School of Medicine, Dentistry and Biomedical Science, Queen's University Belfast, Belfast, United Kingdom; Universidade Federal do Rio de Janeiro (UFRJ), Instituto de Biofísica da UFRJ, Brazil

## Abstract

Ocular neovascularisation is a pathological hallmark of some forms of debilitating blindness including diabetic retinopathy, age related macular degeneration and retinopathy of prematurity. Current therapies for delaying unwanted ocular angiogenesis include laser surgery or molecular inhibition of the pro-angiogenic factor VEGF. However, targeting of angiogenic pathways other than, or in combination to VEGF, may lead to more effective and safer inhibitors of intraocular angiogenesis. In a small chemical screen using zebrafish, we identify LY294002 as an effective and selective inhibitor of both developmental and ectopic hyaloid angiogenesis in the eye. LY294002, a PI3 kinase inhibitor, exerts its anti-angiogenic effect in a dose-dependent manner, without perturbing existing vessels. Significantly, LY294002 delivered by intraocular injection, significantly inhibits ocular angiogenesis without systemic side-effects and without diminishing visual function. Thus, targeting of PI3 kinase pathways has the potential to effectively and safely treat neovascularisation in eye disease.

## Introduction

### Ocular Diseases and Neovascularisation

Functional damage to existing blood vessels and inappropriate development of new blood vessels are hallmarks of prominent forms of blindness. In diabetic retinopathy (DR), which affects ∼150 million patients, neovascularisation of vessels in the adult retina occurs [*1*]. In age-related macular degeneration (AMD), which is estimated to affect ∼8 million US patients and ∼20 million patients in US plus Europe, neovascularisation of capillaries in the choroid layer adjacent to the retina occurs [*2*]. Ocular neovascularisation associated with retinopathy of prematurity (ROP) arises when premature infants are placed into high oxygen to alleviate respiratory distress. As a consequence, concomitant regression of the lens-associated hyaloid vasculature and growth of the nascent retinal vasculature ceases. When returned to normoxia, the infant retina becomes hypoxic leading to neovascularisation, persistent hyaloid vasculature and eventual blindness [3]. Thus, a therapeutic goal for DR and AMD is to develop agents that inhibit ocular neovascularisation and/or stabilise existing vessels, without diminishing visual function.

### Current Ocular Angiogenesis Treatments

Current interventions for resolving inappropriate growth of new vessels in the eye include laser treatment or molecular therapies targeted to vascular endothelial cell growth factor (VEGF). Although the therapeutic mechanism of action is not fully understood, laser photocoagulation surgery can halt neovascularisation and reduce macular oedema [*1*], [*4*]. However, success is partial, requires multiple treatments and side effects include cataracts, hemorrhage, retinal detachment and visual field loss.

Variations of VEGF molecular therapy to inhibit ocular neovascularisation include antibodies, siRNAs or small molecule inhibitors [*5*]–[*10*]. Those in clinical use are antibodies targeted to VEGF (*e.g*. bevacizumab (Avastin®) and randizumab (Lucentis®)) which delay the growth of new vessels and delay vision loss. Treatment also requires intraocular injection by retinal specialists, repeat administrations and patient sedation. Concerns regarding the development of siRNAs to VEGF have also arisen as they may act non-specifically [*7*]. More general concerns regarding the strategy of solely targeting VEGF include, the observed compensatory up-regulation of other pro-angiogenic factors which decrease therapeutic efficacy, and the potential to cause retinal neurodegeneration by inhibiting VEGF's neuroprotectant function [*11*], [12**].

### Alternative Anti Angiogenic Targets

Targeting angiogenic molecules other than, or in addition to VEGF, may reveal more effective and safer inhibitors of intraocular neovascularisation. Potential targets include growth factors (*e.g.* angiopoietin, FGF, HGF, IGF-1, PDGF-B, PlGF), chemokines (*e.g.* IL8, SDF1, G-CSF), receptors (*e.g.* CXCR1, FGF-R, PlGFR, PDGFR, Tie-receptors), intracellular mediators (*e.g*. c-kit kinase, PI3 kinase, PKC) and extracellular mediators (*e.g*. integrins, cadherins) [13].

Several drugs which do not selectively target VEGF have indeed shown anti-angiogenic efficacy in eyes. Pazopanib, PKC412 and TG100572 are small molecule inhibitors that each targets multiple kinases [*6*], [9], [14**]. Pazopanib (Armala®) which blocks PDGFRs, c-Kit, FGFR and c-fms, and TG100572, which inhibits FGF, PDGF and VEGF, both suppress choroidal neovascularisation in mouse models [*6*], [*9*]. TG100572 has improved delivery features as it is effective following local administration as eye drops [*6*]. PKC412 blocks PKC, VEGF-R, PDGF-R and SCF-R isoforms, and in diabetics is reported to reduce macular oedema [*14*]. However, liver toxicity was observed following oral administration.

Another approach is to selectively target a distinct pro-angiogenic molecule. Indeed recent studies report that the broad-spectrum PI3K inhibitor LY294002 and the αvβ3/αvβ5 integrin antagonist EMD478761 suppress retinal or choroidal neovascularisation following intraocular injection in rodents [*15]*, [*16*]. A combination of both approaches involves targeting multiple foci of pro-angiogenic pathways with several selective inhibitors. Dorrell *et al.* report that combination angiostatic therapy with a VEGF aptamer, an integrin antagonist and a proteolytic fragment of tryptophan tRNA synthetase inhibits ocular angiogenesis [*11*].

### Zebrafish Intraocular Vasculature and Chemical Screens

We recently demonstrated that the inner retina of zebrafish is nourished by an intricate vascular network which shares many features with human hyaloid and retinal vasculatures [*17*]. Initially this intraocular vasculature presents as a hyaloid vasculature more tightly associated with the lens which in adults has migrated to firmly attach to the inner retina. We identified a cohort of genes necessary for normal hyaloid vasculature formation in zebrafish [*17*]. In addition to amenability towards genetic screens, zebrafish are also suitable for pharmacological screens [*18*], [*19*]. In relation to intraocular vasculature, Cao *et al.* recently show that exposure of adult zebrafish to hypoxia combined with treatment with a notch signaling inhibitor is sufficient to induce retinal neovascularisation [*20*]. In a random screen of small molecules, Kitambi *et al.* recently identified novel molecules inhibiting hyaloid angiogenesis in zebrafish larvae [*21*].

### Summary

Here, we conduct a targeted screen of known regulators of angiogenesis to identify drugs that inhibit developmental angiogenesis in the zebrafish eye. LY294002, a small molecule inhibitor of PI3 kinase was identified as an effective inhibitor of developmental angiogenesis in the eye. LY294002 inhibits new vessel growth without any apparent effect on existing vessels. The anti-angiogenic effect of LY294002 is dose-dependent and can be phenocopied using an inhibitor to AKT, which itself is known to act downstream of PI3K. Importantly, intraocular administration of LY294002 selectively inhibits angiogenesis in the eye but does not diminish visual function.

## Methods

### Ethics Statement

All experiments were carried out under ethical approval granted by the UCD animal research ethics committee (http://www.ucd.ie/researchethics/arec.html).

### Drug Treatment

*Tg(fli1:EGFP)* zebrafish were maintained according to standard procedures on a 14 h light/10 h dark cycle at 28°C. Embryos were obtained by natural spawning and developmental stages established by time and morphological criteria. At 24 hpf, 5 embryos per well were placed in 400 µl of Embryo Medium/1% DMSO and incubated with drug at 28°C on a 14 h light/10 h dark cycle [*22*]. Larvae were euthanised, and fixed in 4% PFA at 4°C overnight before analysis.

### Quantification of Primary Branch Number

**Figure 1:**
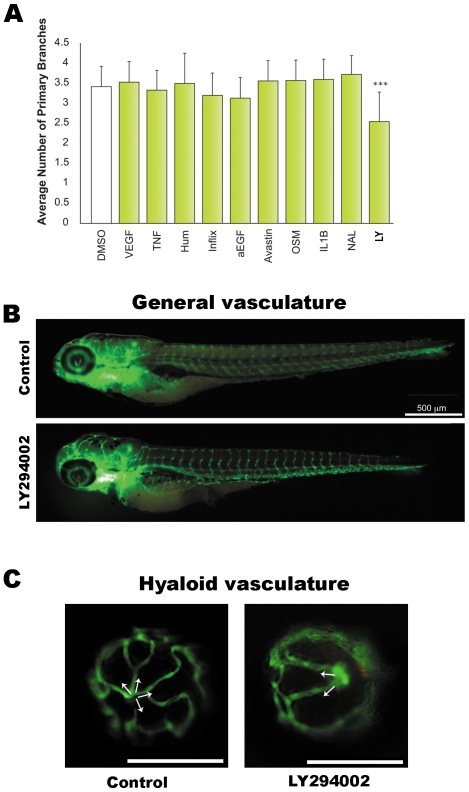
Screens reveal LY294002 to inhibit developmental angiogenesis in the eye. **A**. Known regulators of vasculature formation were screened for effects on developmental angiogenesis of the hyaloid vasculature in vivo. 1 day post fertilisation (dpf) *Tg(fli1:EGFP)* larvae were treated with the indicated drugs and the number of primary branches of hyaloid vessels determined at 5 dpf. The drugs used are: recombinant VEGF (VEGF), 50 ng/ml; Tumor Necrosis Factor (TNF), 20 ng/ml; Adalimumab - Humira® (Hum), 5 µg/ml; Infliximab (Inflix), 5 µg/ml; anti-EGF (α-EGF), 5 µg/ml; Bevacizumab- Avastin®, 5 µg/ml; Oncostatin M (OSM), 10 ng/ml; Interleukin 1 beta (IL-1B), 10 ng/ml; Nacyselyn (NAL), 10 µM; and LY294002 (LY), 10 µM. The data is plotted as the average and standard deviation of 3 replicate experiments. LY294002 results in a statistically significant inhibition of hyaloid vasculature development (***p<0.001, t-test). **B–C**. Fluorescent images illustrating the integrity of GFP-positive blood vessels at 5 dpf in LY294002-treated and DMSO-treated controls. No differences are observed in the morphology of the trunk vessels (B), whereas the hyaloid vessel branching pattern is abnormal and the branch number is reduced (C). White arrows in C indicate the primary branches radiating from the optic disc at the back of the lens.

Prior to analysis of the intraocular vasculature, control- and treated-larvae were screened under an Olympus SZX16 microscope for general malformations. The overall patterning of the fin, gut and intersegmental vasculature was examined. Larval lenses from right eyes were dissected, transferred to depression slides and observed by epi-fluorescence microscopy using an Olympus SZX16, or placed in petri-dishes with cover-slip bottoms for confocal microscopy using a Zeiss UV510 META LSM. To orientate, lenses were embedded in 10% methyl-cellulose and manipulated with a 0.5 mm tungsten needle. Number of primary branches radiating from optic disc at the back of lens was determined for each specimen (see white arrows in Fig. 1).

### Regenerative Angiogenesis Assay

Adult *Tg(fli1:EGFP)* zebrafish were anesthetised in 0.02% Tricaine (Sigma) and caudal, dorsal or anal fins amputated with a scalpel below the second branch points. Fish were incubated in tank water or tank water/10 µM LY294002 (Sigma) for 9 days, euthanised in 0.04% Tricaine, decapitated and fixed in 4% PFA (Sigma) for 24 hours at 4°C. Fins were mounted on glass slides in VectaShield (Vector) and visualised on an Olympus S2X12 fluorescent microscope. Images were acquired using a Hamamatsu ORCA-ER camera with OpenLab capturing software and adapted for publication in Photoshop CS2 9.02.

### Electroretinography

Electroretinograms (ERGs) were performed on *Tg(fli1:EGFP)* zebrafish treated from 2–5 dpf with 10 µM LY294002 (n = 15) or 1% DMSO vehicle (n = 13). ERGs were performed *in vivo* during the afternoon as described by Fleisch *et al.*, with a few modifications [*23*]. Larvae were paralysed with 0.5 mg/ml mivacurium chloride (Mivacron®) and the recording electrode filled with 0.9% saline. A Kodak projector containing a 300W halogen light source was used for light stimulation. The unattenuated irradiance stimulus was 2.8×10^3^ µW/cm^2^. Three optical density filters were used to produce flash intensities of −3.0 log, −2.0 log and −1.0 log together with the unattenuated stimulus, −0 log. Flash duration was 100 milliseconds for all intensities, with an interstimulus interval of between 10 seconds and 1 minute, depending on flash intensity. To digitise and visualise data, a NIDAQ 6024E board (National Instruments) and Windows Whole Cell Programme software (WinWCP V3.9.1; courtesy of John Dempster, Strathclyde University, Scotland) were used. Data shown are signal averages of 2–10 responses. The a-wave was measured from baseline to the trough of the a-wave, and the b-wave was measured from the trough of the a-wave to the peak of the b-wave. Raw data from the sample groups were compared using an independent 2-sample t-test with unequal variances.

### Visual Behaviour Assay

Visual function was assessed by the optokinetic-response (OKR) assay [*24*]. Briefly, 5 dpf larvae in Embryo Medium/9% methylcellulose were placed inside a black and white striped drum. The drum was rotated for 30 seconds in clockwise then counterclockwise directions and the number of eye saccades counted.

### Retinal Light Microscopy

Larvae were euthanised with a lethal dose of Tricaine and fixed in 0.1 M Sorensen phosphate buffer (pH 7.3)/4% PFA/2.5% glutaraldehyde overnight at RT. Larvae were post-fixed in 0.1 M Sorensen phosphate buffer/1% osmium tetroxide for 1 hour at RT, dehydrated in ascending concentrations of alcohol to 100% and embedded in Epon resin using standard methods [*25*]. 1 µm sections were stained for 20 seconds with toluidine-blue, and examined by light microscopy using a Leica DMLB bright field illumination microscope and a Leica DFC 480 camera.

### Intraocular Drug Treatments

*Tg(fli1:EGFP)* larvae were anesthetised with 0.02% Tricaine, dechorionated and transferred to 3 cm Petri dishes containing 1 ml of cold-liquid CyGEL™ (Biostatus). Larvae were immobilised in CyGEL at room temperature with the left eye facing upwards for injection **(Fig. S1)**. Immobilised larvae were injected in the left eye at 1 or 2 dpf with 10 µM LY294002 or 1% DMSO diluted in ddH_2_O/1% FastRed tracer dye using heat pulled glass capillaries (bore diameter OD/ID: 1/0.58 mm). After intraocular injection, larvae were retrieved by placing the dish on a chilled ice pad to melt the CyGEL and immediately transferred to Embryo Medium at RT for recovery. Larvae were maintained at 28°C until 5 dpf and hyaloid vasculature analysed as described above.

## Results

### LY294002 Inhibits Developmental Angiogenesis of the Eye

To identify drugs that significantly alter development of new blood vessels in the eye, we performed a small pharmacological screen in zebrafish. The drugs screened (**Table S1**) are known to control growth factor-, cytokine- or inflammatory-mediated angiogenesis in other tissues, and several are used clinically to treat AMD, cancer or rheumatoid arthritis [26]–[28]. *Tg(fli1:EGFP)* larvae which specifically express EGFP in all blood vessels [*29*] were immersed in drug from 1–5 days post fertilisation (*dpf*) and the integrity of the hyaloid vasculature determined by fluorescent microscopy of dissected lenses.

None of the growth factors, cytokines or antibodies affect the number of primary branches of hyaloid vasculature **(** **Fig. 1A** **)**. However, the small molecule drug LY294002 significantly reduces the number of primary branches of hyaloid vasculature, indicative of anti-angiogenic activity **(** **Fig. 1A** **)**. Analysis of wholemount larvae suggests that LY294002 treatment has no significant effect on the intersegmental or trunk vessels **(** **Fig. 1B** **)**, which develop earlier than the hyaloid vasculature [*17*], [*29*]. Confocal analysis clearly demonstrates that treatment with 10 µM LY294002 reduces the branch number and patterning of the hyaloid vasculature **(** **Fig. 1C** **)**. This result suggests that the PI3K inhibitor, LY294002, inhibits the formation of new vessels but does not reduce the number of existing vessels systemically.

### Dose- and Time-Dependent Inhibition of Intraocular Angiogenesis by LY294002

**Figure 2:**
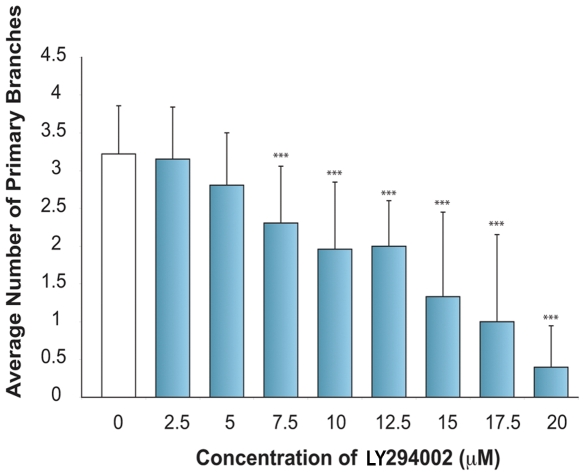
LY294002 results in dose-dependent inhibition of intraocular angiogenesis. 1 dpf *Tg(fli1:EGFP)* larvae were treated with LY294002 at concentrations ranging from 2.5–20 µM and the number of primary branches of hyaloid vessels determined at 5 dpf. The data graphed is plotted as the average and standard deviation of 3 replicate experiments. LY294002 results in a statistically significant inhibition of hyaloid vasculature development (***p<0.001, t-test).

Treating larvae from 1–5 dpf, with a range of LY294002 concentrations, reveals a dose-dependent inhibition of hyaloid vessel angiogenesis with an IC_50_ of ∼12 µM **(** **Fig. 2** **)**. At concentrations from 7.5–12.5 µM LY294002, statistically significant inhibition of hyaloid angiogenesis is observed in the absence of gross morphological defects. Larvae incubated in 10 µM LY294002 occasionally display mild oedema indicative of systemic side effects. At concentrations of 17.5 µM LY294002 and above, systemic toxic effects including oedema, bent body axis and craniofacial abnormalities are observed. Thus, 10 µM LY294002 was utilised for all additional experiments.

**Figure 3:**
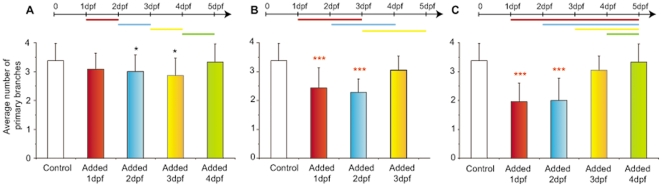
Time-dependent inhibition of intraocular, developmental angiogenesis by LY294002. *Tg(fli1:EGFP)* larvae were treated with 10 µM LY294002 for the indicated time frames and the number of primary branches of hyaloid vessels determined at 5 dpf. The data is plotted as the average and standard deviation of 3 replicate experiments. 10 µM LY294002 is added for 24 hours (**A**) or 48 hours (**B**) starting at 1, 2, 3 or 4 dpf. In **C** 10 µM LY294002 is added at 1 dpf for 96, 72, 48 or 24 hours. In any experimental variation, the presence of LY294002 from 2–3 dpf is sufficient to result in a statistically significant inhibition of developmental angiogenesis of hyaloid vessels (*p<0.05; ***p<0.001, t-test).

To determine if LY294002 inhibits hyaloid angiogenesis during a specific developmental time-frame, 1–4 dpf *Tg(fli1:EGFP)* larvae were treated for 24, 48, 72 or 96 hours, and hyaloid vasculature analysed at 5 dpf **(** **Fig. 3** **)**. If LY294002 is added to 1 or 4 dpf larvae for 24 hours, development of the hyaloid branches is unaffected **(** **Fig. 3** **A & C)**. However, the presence of LY294002 from 2–3 dpf is sufficient to result in a statistically significant inhibition of hyaloid angiogenesis. These results are consistent with LY294002 blocking developmental angiogenesis of new hyaloid vessels but exerting no effect on existing intraocular vessels.

### The PI3K-Akt Pathway Controls Hyaloid Angiogenesis

**Figure 4:**
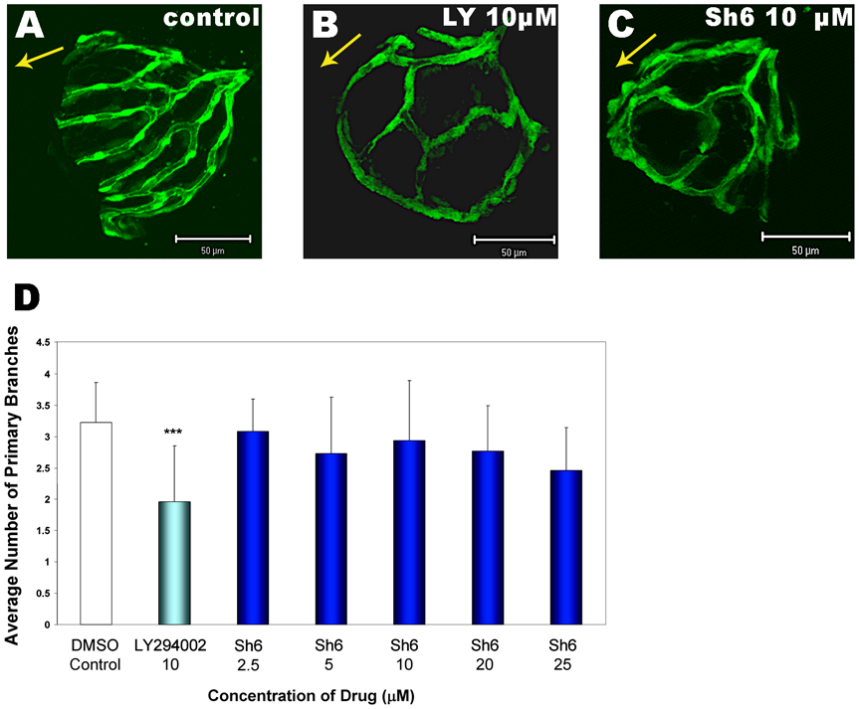
The Akt inhibitor SH6 phenocopies the PI3K inhibitor LY294002. Comparison of hyaloid vasculature patterning in DMSO-treated control larvae (**A**), 10 µM LY294002 treated larvae (**B**) and larvae treated with the Akt inhibitor SH6 at 10 µM (**C**). All larvae are from the *Tg(fli1:EGFP)* line and were treated from 1–5 dpf. Like LY294002, SH6 affects angiogenesis of the hyaloid vessels. Abnormal patterning of the hyaloid vasculature is observed with SH6 (**C**) resembling LY294002-treated larvae (**B**), though no significant reduction in primary vessel number is observed with SH6 (**D**). The data shown in panel D is plotted as the average and standard deviation of 3 replicate experiments (***p<0.001, t-test).

To corroborate the anti-angiogenic activity of LY294002 on hyaloid vasculature being mediated via inhibition of PI3K signalling, we selectively inhibited the activity of Akt/PKB, a downstream target of PI3K [30]. Analysis of hyaloid vessels at 5 dpf demonstrates that SH6, a PKB/Akt inhibitor, can phenocopy LY294002. Both drugs at 10 µM, result in a striking difference in the patterning of hyaloid vessels **(** **Fig. 4** **A–C)**. Interestingly, LY294002, but not SH6, also significantly reduces the number of primary hyaloid vessels **(** **Fig. 4** **D)**.

### LY294002 Attenuates Ectopic and Regenerative Angiogenesis

**Figure 5:**
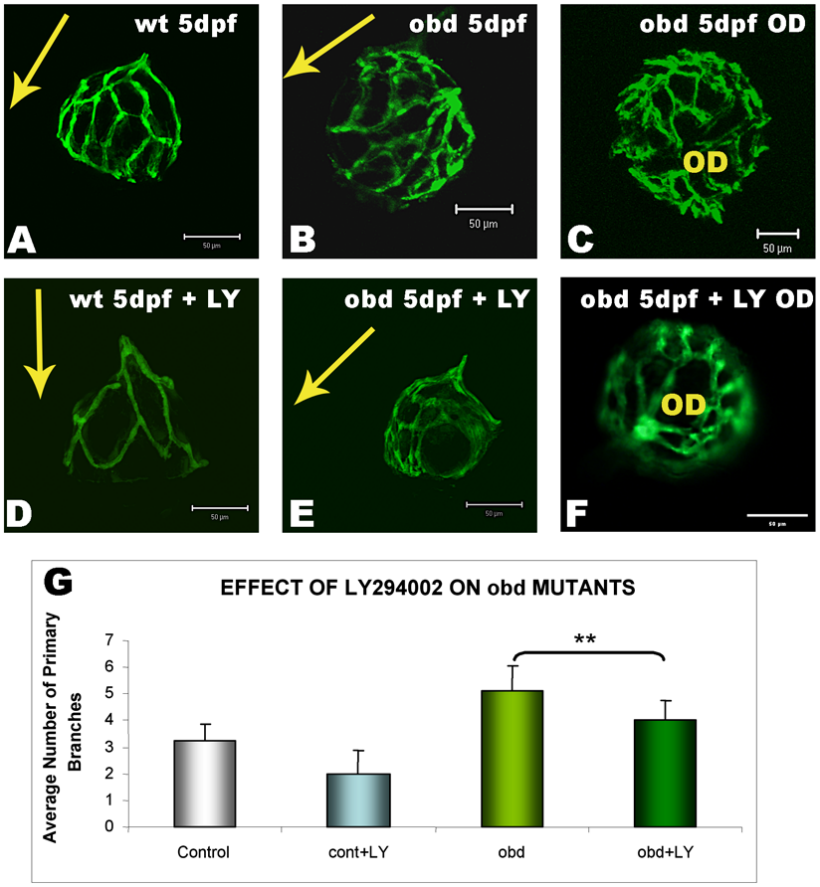
LY294002 attenuates ectopic developmental angiogenesis in *obd* mutants. Wildtype *Tg(fli1:EGFP)* larvae (wt) or *out of bounds* mutants in the *Tg(fli1:EGFP)* background (*obd*) were treated with 10 µM LY294002 from 1–5 dpf before dissecting lenses and analysing hyaloid vasculature development. LY294002 inhibits normal developmental angiogenesis in wildtype *Tg(fli1:EGFP)* larvae as seen by the reduced vessel patterning in lateral views of the lens *(* ***A, D*** *)*. LY294002 also inhibits the extraneous hyaloid branch patterning (**B**) and branch number (**C**) observed in *obd* mutants as observed by reduction of branch patterning to near normal levels *(* ***E and F*** *)*. Arrows depict the orientation of the lens, pointing in the direction from the optic disk to lens. **G**) Quantification of the reduction in the extraneous primary hyaloid branches number in LY294002-treated *obd* mutants. The data is plotted as the average and standard deviation of larvae (*obd^−/−^* n = 7, *obd^−/−^* treated: n = 8) from 2 independent experiments (***p<0.001, t-test). *OD: Optic Disc*.

The ability of LY294002 to inhibit extraneous angiogenesis was determined in *out of bounds* (*obd)* mutants. As a consequence of a recessive mutation in plexin D, *obd^−/−^* larvae present with ectopic developmental angiogenesis [*17*], [*31*]. Treatment of *obd^−/−^* larvae with 10 µM LY294002 inhibits ectopic hyaloid angiogenesis and can reduce branch patterning to near wild type levels **(** **Fig. 5** **)**.

**Figure 6:**
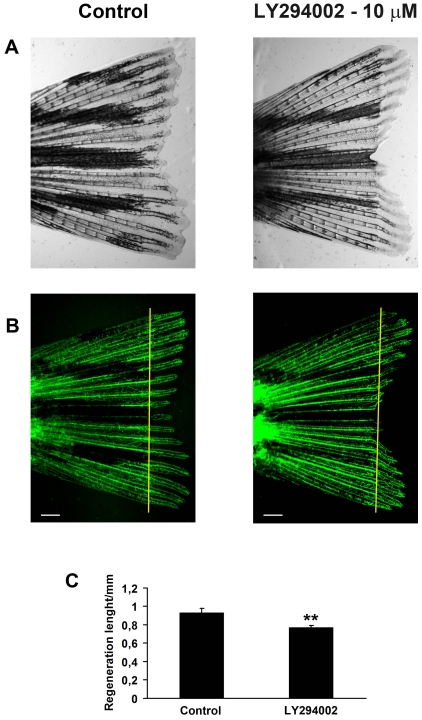
LY294002 attenuates Regenerative Angiogenesis in Adult Zebrafish. The caudal fins of adult Tg(*fli1:EGFP*) zebrafish were amputated and left to regenerate in either tank water or tank water supplemented with 10 µM LY294002. Bight field micrographs (**A**) or fluorescent micrographs (**B**) of representative fins at 9 days post-amputation are shown. The maximal, average blood vessel length in the regenerated tissue was quantified at the third fin ray in either fin lobe and the results are depicted in (**C**). LY294002 significantly inhibits regenerative angiogenesis in the tail fin (** p<0.01, t-test). Yellow lines in B indicate the amputation plane. n = 8 fin rays in 4 fish. Scale bars = 500 µm.

The anti-angiogenic efficacy and safety of LY294002 in the context of adult tissues was determined using a fin regeneration assay [*32*]. The extent of angiogenesis in regenerating fins was determined in adult fish following caudal, dorsal or anal fin clipping. Compared to controls, LY294002 significantly inhibits regenerative angiogenesis **(** **Fig. 6** **and Fig S2)**. LY294002 also inhibits regeneration length, which is reported to be angiogenesis-dependent [32] **(** **Fig. 6** **and Fig S2)**. Otherwise, no toxic effects were observed, and LY294002- and control-treated adults were indistinguishable in relation to behaviour, ventilation rate and swimming patterns.

### LY294002 Inhibits Retinal Angiogenesis without Affecting Retinal Function

**Figure 7:**
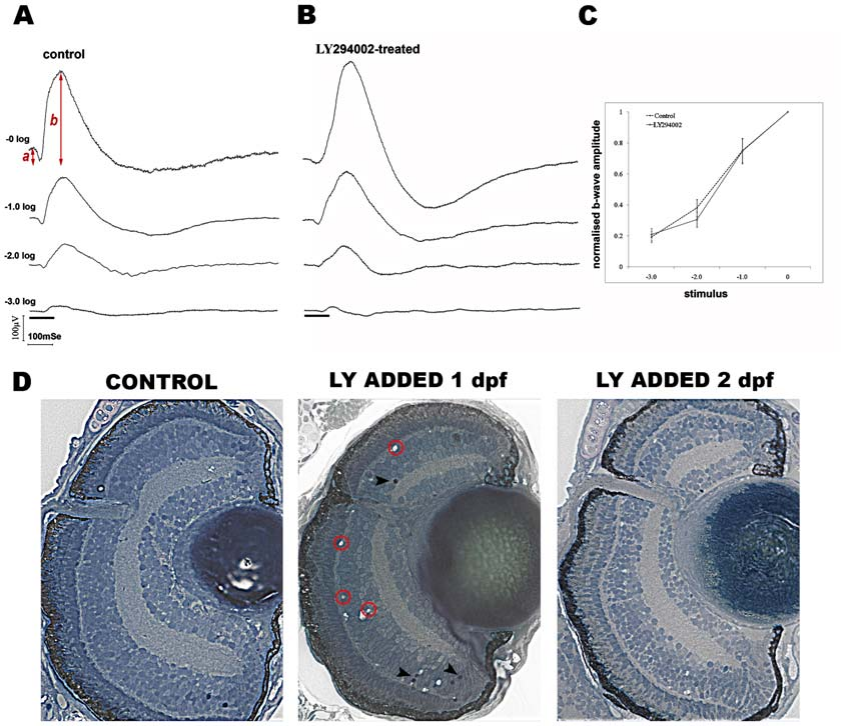
LY294002 inhibits retinal angiogenesis without affecting retinal function. **A–C**) Electroretinography of control (n = 13) and LY294002-treated (n = 15) zebrafish at 5 dpf. Shown is representative data from control (**A**) and LY294002-treated (**B**) larval zebrafish in response to an intensity series of white light stimulation. The stimulus flash was attenuated from 3 log units to unattenuated. The black bars at the bottom represent stimulus duration. The two groups show similar intensity response curves for the b-wave (**C**). To generate the intensity response curve, the average response at the maximum flash intensity was set to 1 and the average response at attenuated flash intensities was normalised to this (average ± SEM). **D**) Histological sections of zebrafish eyes from 5 dpf larvae treated with LY294002 from 1–5 dpf or 2–5 dpf. Retinal histology appears normal in fish treated from 2–5 dpf showing lamination, presence of optic nerve and similar size/shape to controls. In larvae treated from 1–5 dpf sparse apoptotic nuclei (arrowheads) and tissue vacuoles are observed *(Magnification 400×)*.

The electroretinogram (ERG) is a diagnostic method for quantifying outer retinal function. ERGs from larvae treated with LY294002 or vehicle from 2–5 dpf show equivalent responses at 4 flash intensities **(** **Fig. 7A–C** **)**. At maximum flash intensity, the a-wave measure of photoreceptor hyperpolarisation has amplitudes of −28±5.8 µV or −31±3.5 µV, and implicit times of 61±7.6 milliseconds (ms) or 68±7.7 ms in control and drug-treated larvae, respectively. Similarly, the b- wave which reflects signal transmission to bipolar cells has amplitudes of 199±20.5 µV or 208±28 µV and implicit times of 157±10.5 ms or 169±10.1 ms in control and drug-treated larvae, respectively **(** **Fig. 7A–C** **)**. In agreement, the retinal histology of larvae treated with LY294002 from 2–5 dpf is indistinguishable from controls. Both samples consist of laminated retinas with prominent optic nerves and transparent lenses **(** **Fig. 7D** **)**.

Retinal histology in larvae treated earlier from 1–5 dpf does feature apoptotic nuclei and tissue vacuolation **(** **Fig. 7D** **)**. This is consistent with a loss of visual function in larvae treated with LY294002 for just 24 hours beginning at 1 dpf and suggests a role for PI3K in early retinal development, independent of angiogenesis. In summary, treatment with LY294002 can block hyaloid angiogenesis with no detrimental effect on retinal function.

### Intraocular LY294002 Selectively Inhibits Angiogenesis and not Visual Behaviour

**Figure 8:**
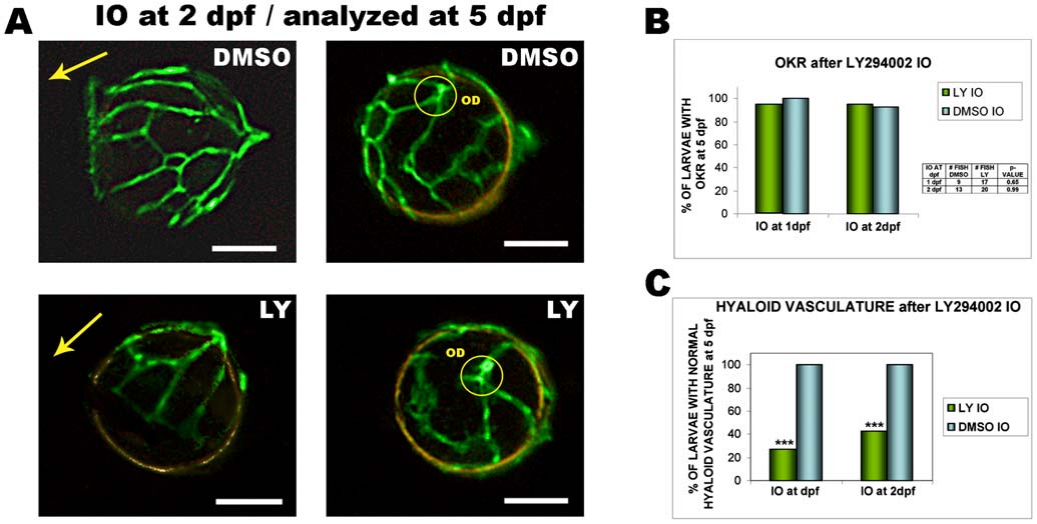
Intraocular injection of LY294002 inhibits angiogenesis but not visual function. **A**) The hyaloid vasculature of 5 dpf larvae intraocularly injected with 10 µM LY294002 at 2 dpf shows abnormal patterning and reduced numbers of primary branches compared with controls injected with 1% DMSO (left images are lateral views, and right images are dorsal views of lenses. *OD: optic disc; scale bars: 50 µm*) **B**) Graph of the percentage of 5 dpf larvae with optokinetic responses (OKR) following intraocular injection of LY294002 at 1 or 2 dpf. The Fisher test shows no statistically significant difference in visual function when LY294002 is injected intraocularly at 1 or 2 dpf (p values shown on table) **C**) Graph showing percentage of 5 dpf larvae with normal hyaloid vasculature after injection of LY294002 at 1 or 2 dpf. Intraocular LY294002 at 1 or 2 dpf, significantly inhibits hyaloid angiogenesis. Data graphed from 3 independent experiments; LY294002 at 1 dpf, n = 17; 1% DMSO at 1 dpf, n = 9; LY294002 at 2 dpf, n = 26; 1% DMSO at 2 dpf, n = 13 (***p<0.001, Chi-Square test).

Finally, we determined if localised delivery of LY294002 can inhibit intraocular angiogenesis, and do so without systemic side-effects, or effects on retinal function. Compared to larvae injected with vehicle alone, larvae injected with 10 µM LY294002 do display significant inhibition of hyaloid vessel angiogenesis **(** **Fig. 8** **A, C)**. Unlike the mild oedema observed in larvae treated systemically with LY294002, intraocular injection of LY294002 has no obvious systemic side-effects. Visual function in larvae injected intraocularly with LY294002 was analysed by the optokinetic response (OKR) [*24*]. In this assay, fish with normal visual function track rotating black and white stripes via reproducible saccades. No difference is observed in the OKR response of fish injected intraocularly with vehicle alone or with LY294002 **(** **Fig. 8B** **)**. These results indicate that intraocular administration of LY294002 can selectively block angiogenesis in the eye without detrimental effects on visual function.

## Discussion

### Summary

We report here evidence demonstrating that the small molecule inhibitor LY294002 can be an effective and safe inhibitor of ocular angiogenesis *in vivo*. LY294002 inhibits normal and extraneous developmental angiogenesis in the zebrafish eye, in dose- and time-dependent manners. Systemic administration of LY294002 results in oedema and locomotor activity defects are indicated (data not shown). However, intraocular administration of LY294002 can selectively inhibit intraocular angiogenesis without affecting retinal morphology or visual function.

### LY294002- An Ocular Anti-Angiogenic

Does LY294002 or its cellular target have potential for clinical use as an ocular anti-angiogenic? Our data demonstrates that LY294002 is an effective inhibitor of hyaloid vasculature angiogenesis in the zebrafish eye. In addition, recent studies report that intraocular administration of LY294002 reduces unwanted retinal and choroidal neovascularisation in murine models [*15*], [*16*]. The mouse studies did not address potential effects of LY294002 on retinal function, but our study shows that short-term treatment with LY294002 can have no effect on ocular morphology or visual physiology. LY294002 is most likely exerting its anti-angiogenic activity by inhibiting the PI3K signalling pathway, which is known to mediate angiogenesis [*30*]. Indeed, LY294002 is a classic, broad-spectrum inhibitor of phosphoinositide 3-kinases (PI3K) [*33*]. The PI3K enzyme family act as second messengers during signal transduction events associated with diverse cellular activities, including migration, proliferation and differentiation [*30*]. Recent evidence suggests that LY294002 also inhibits casein kinase, GSK3β and mTOR (a PI3K-related kinase) [*34*]. LY294002, however, was previously excluded from clinical development due to toxicity issues. These may arise from the broad-spectrum of PI3Ks inhibited, off-target effects or systemic administration.

### Refined Targeting of PI3K Pathways

Our data suggests that LY294002 can have selective anti-angiogenic effects in the eye following intraocular administration. No perturbations of retinal function or morphology are observed. Future studies will need to determine if the lack of toxicity is due to local administration to the eye, species difference or short-term treatment.

However, instead of using a broad-spectrum PI3K inhibitor like LY294002, it should also be possible to selectively interfere with the subset of PI3K signals associated with pathological angiogenesis in the eye. The development of isoform-selective PI3K inhibitors has already led to their approval for clinical trials in cancer [*35*], [*36*]. The PI3K family is divided into several classes based on protein structure and substrate targets. The signal transduction mechanism is best understood for Class I PI3Ks [*30*]. These are heterodimers, comprising of one of four p110 catalytic subunits (*α, β, δ* or *γ*) plus a regulatory subunit. Growth factor or G protein-coupled receptor activation recruits the regulatory subunit of PI3K resulting in activation of the catalytic subunit. Subsequent conversion of PI(4,5)P_2_ to PI(3,4,5)P_3_ promotes the recruitment of proteins exemplified by AKT to the plasma membrane. AKT is phosphorylated by phosphoinositide-dependent protein kinases and targets proteins involved in angiogenesis, apoptosis, cell growth and glucose uptake [*37*], [*38*].

Analysis of p110*δ* and *γ* reveals specific roles for these isoforms in the immune system. Indeed, IC87114, an inhibitor selective for p110*δ*, was subsequently found to inhibit a form of leukaemia and is now in clinical trial [*39*]. Similarly, genetic analyses demonstrate that the p110*α* isoform is selectively required for angiogenesis [*40*]. To our knowledge no p110*α*-selective inhibitor has gone on to clinical development. However, several potential p110*α* selective inhibitors have been reported [*35*], [*39*]. Our data with LY294002 strongly support testing the efficacy and safety of p110*α*-selective PI3K inhibitors in models of ocular neovascularisation.

An alternative approach to isoform-selective PI3K inhibitors is the use of a modified version of LY294002. Until recently, it was generally considered that broad-spectrum PI3K inhibitors would not have clinical use due to toxicity arising from inhibition of physiological signalling. However, SF1126 is a modified version of LY294002 in clinical development for cancer. It consists of LY294002 linked to a peptide sequence that targets it to the vasculature [*41*]. Future studies are warranted to test the efficacy and safety of this prodrug in models of ocular angiogenesis.

### Screens for Ocular Anti-Angiogenics in Zebrafish

Here we provide further *proof-of-principle* that chemical screens in zebrafish can uncover novel anti-angiogenic drugs for the eye. The screens are relatively simple with 5 larvae per well incubated from 1–5 days post-fertilisation with an experimental drug. Assessment of ocular angiogenesis is facilitated by the using the *Tg(fli1:EGFP)* line and a fluorescent dissecting microscope. The limiting step is the labour-intensive dissection of lenses but this may be overcome with improvements in imaging *in vivo*. Kitambi *et al*. recently identified several drugs that perturb intraocular angiogenesis *in vivo* [*21*] and our study demonstrates how such drugs can be further characterised to address specificity and safety. Finally, our screens appear most suited to stable small molecule chemicals as antibodies or recombinant proteins had no effect, most likely due to poor bioavailability in the eye or degradation.

### Future Perspectives

In summary, we provide evidence that a broad-spectrum PI3K inhibitor can attenuate ocular angiogenesis without perturbing visual function. Thus, there can be cautious optimism about the potential clinical use of PI3K inhibitors to treat unwanted ocular neovascularisation. This could involve administration of an isoform-specific inhibitor or a vasculature-targeted prodrug, either as monotherapies or part of combination angiostatic therapies.

## Supporting Information

Figure S1 Intraocular Injection of LY294002 in zebrafish larvae. A) ∼5 dechorionated zebrafish larvae (48 hpf in the figure) are immobilized in CyGEL at RT and oriented with left eye facing upwards. Dechorionation is extremely important as non-dechorionated larvae collapse in contact with CyGEL. B) Intraocular injection (0.1–0.2 µl/eye) is performed with a heat pulled glass capillary. C) After intraocular injection the Petri dish is placed on a chilled ice pad to melt CyGEL and larvae are transferred gently but immediately to a plate with RT embryo medium for recovery.
(4.36 MB TIF)

Figure S2 Regenerative angiogenesis in zebrafish dorsal or anal fins is inhibited by LY294002. The dorsal (A, C and E) or anal fins (B, D and F) of adult *Tg(fli1:EGFP)* zebrafish were amputated and left to regenerate in either tank water or tank water supplemented with 10 µM LY294002. Bight field micrographs (A and B) or fluorescent micrographs (C and D) of representative fins at 9 days post amputation are shown. The maximal, average blood vessel length in the regenerated tissue was quantified at the third fin ray from either side of each image and the results are depicted in (E and F). LY294002 significantly inhibits regenerative angiogenesis in both dorsal and anal fins (***p<0.001; t-test). Yellow lines in C and D indicate the amputation planes. n = 8 fin rays in 4 fish. Scale bars = 500 µm.
(4.74 MB TIF)

Table S1 Pro- and Antiangiogenic drugs screened for effects on angiogenesis of the zebrafish hyaloid vasculature.
(0.03 MB DOC)
