# Supplementary figures and images for: Selective Inhibition of Retinal Angiogenesis by Targeting PI3 Kinase
Source: PLoS One. 2009 Nov 17;4(11):e7867. doi: 10.1371/journal.pone.0007867 (PMC2773410; doi:10.1371/journal.pone.0007867)

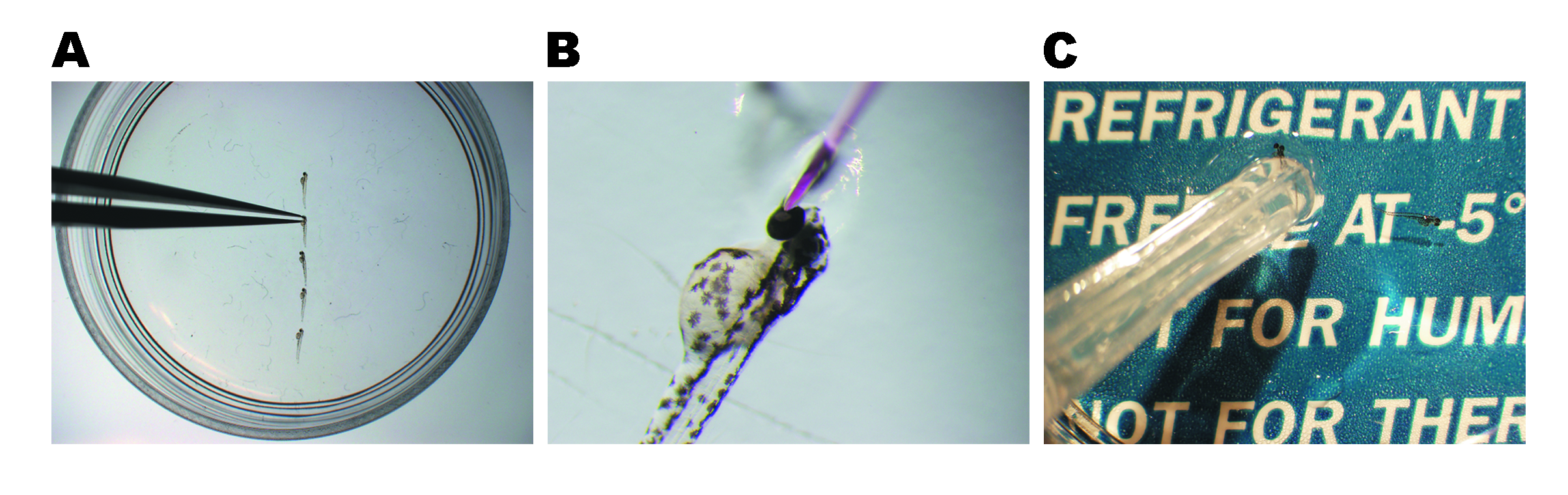

Supplement: Figure S1 — Intraocular Injection of LY294002 in zebrafish larvae. A) ∼5 dechorionated zebrafish larvae (48 hpf in the figure) are immobilized in CyGEL at RT and oriented with left eye facing upwards. Dechorionation is extremely important as non-dechorionated larvae collapse in contact with CyGEL. B) Intraocular injection (0.1–0.2 µl/eye) is performed with a heat pulled glass capillary. C) After intraocular injection the Petri dish is placed on a chilled ice pad to melt CyGEL and larvae are transferred gently but immediately to a plate with RT embryo medium for recovery. (4.36 MB TIF) [file pone.0007867.s001.tif]

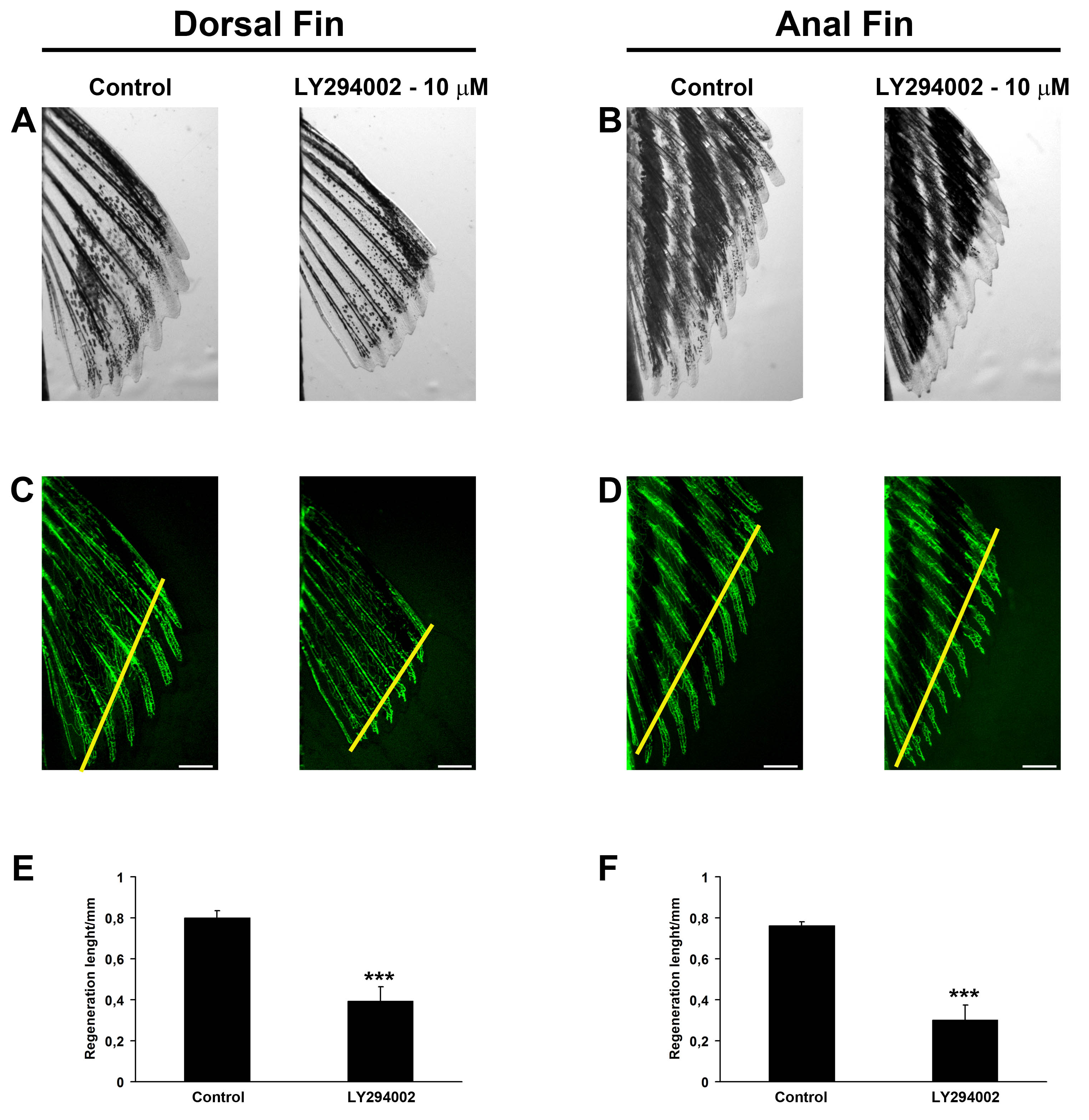

Supplement: Figure S2 — Regenerative angiogenesis in zebrafish dorsal or anal fins is inhibited by LY294002. The dorsal (A, C and E) or anal fins (B, D and F) of adult Tg(fli1:EGFP) zebrafish were amputated and left to regenerate in either tank water or tank water supplemented with 10 µM LY294002. Bight field micrographs (A and B) or fluorescent micrographs (C and D) of representative fins at 9 days post amputation are shown. The maximal, average blood vessel length in the regenerated tissue was quantified at the third fin ray from either side of each image and the results are depicted in (E and F). LY294002 significantly inhibits regenerative angiogenesis in both dorsal and anal fins (***p<0.001; t-test). Yellow lines in C and D indicate the amputation planes. n = 8 fin rays in 4 fish. Scale bars = 500 µm. (4.74 MB TIF) [file pone.0007867.s002.tif]
