# Supplementary material for: Selective Inhibition of Retinal Angiogenesis by Targeting PI3 Kinase
Source: PLoS One. 2009 Nov 17;4(11):e7867. doi: 10.1371/journal.pone.0007867 (PMC2773410; doi:10.1371/journal.pone.0007867)
Supplement: Table S1 — Pro- and Antiangiogenic drugs screened for effects on angiogenesis of the zebrafish hyaloid vasculature. (0.03 MB DOC) [file pone.0007867.s003.doc]

| **DRUG** | **EFFECT** | **ACTIVITY** |
| --- | --- | --- |
| Recombinant Vascular Endothelial Growth Factor (VEGF) | Proangiogenic | Main angiogenic growth factors. Induces vasodilation, vascular permeability, endothelial cell mitosis and migration. |
| Tumor Necrosis Factor (TNF) | Proangiogenic | Activator of NF-κB. Increases vascular permeability |
| Adalimumab (Humira®) | Antiangiogenic | Human monoclonal antibody against TNFα |
| Infliximab (Remicade®) | Antiangiogenic | Mouse-human monoclonal antibody against TNFα |
| anti-Epidermal Growth Factor (aEGF) | Antiangiogenic | Monoclonal antibody against EGF |
| Bevacizumab (Avastin®) | Antiangiogenic | Monoclonal antibody recognizing all isoforms of VEGF |
| Oncostatin (OSM) | Proangiogenic | Cytokine promoting endothelial cell migration |
| Interleukin 1B (IL1B) | Proangiogenic | Mediator cytokine in inflammatory response |
| Nacyselyn (NAL) | Antiangiogenic | NF-κB inhibitor, (modulates pro-inflammation by inhibiting cytokine release) |
| LY 294002 | Antiangiogenic | General PI3K inhibitor |
| Sh6 | Antiangiogenic | AKT Inhibitor in PI3K pathway |
